# The H2Valdien derivatives regulate the epithelial–mesenchymal transition of hepatoma carcinoma cells through the Hedgehog signaling pathway

**DOI:** 10.1515/med-2024-0954

**Published:** 2024-06-20

**Authors:** Xuhui Zhao, Xiangxiang Shao, Xiaomin Huang, Chunyan Dang, Ruilin Wang, Hongling Li

**Affiliations:** The First Clinical Medical College, Gansu University of Traditional Chinese Medicine, 730000, Lanzhou, China; Department of Oncology, Gansu Provincial Hospital, 730000, Lanzhou, China

**Keywords:** H_2_Valdien derivatives, Hh signaling pathway, EMT, hepatoma carcinoma

## Abstract

This research delves into the influence of H2Valdien derivatives on the proliferation, migration, and apoptosis induction in hepatoma carcinoma cells (HepG2, Huh-7, and SMMC-7721), with a specific emphasis on inhibiting epithelial–mesenchymal transition (EMT) through modulation of the Hedgehog (Hh) signaling pathway. Utilizing the cell counting kit-8 method, flow cytometry, TUNEL assay, wound healing, and transwell assays, we observed a dose-dependent growth arrest and apoptosis induction in HepG2, Huh-7, and SMMC-7721 cells. Notably, H2Valdien derivatives exhibited a capacity to reduce migration and invasion, impacting the expression of EMT-associated proteins such as *N*-cadherin, vimentin, and E-cadherin. Mechanistically, these derivatives demonstrated the inhibition of the Hh signaling pathway by inactivating Sonic Hh (Shh) and smoothened proteins. This study underscores the robust antiproliferative and apoptosis-inducing effects of H2Valdien derivatives on hepatoma carcinoma cells and elucidates their regulatory role in EMT through modulation of the Hh signaling pathway, providing valuable insights for potential therapeutic interventions.

## 1 Introduction

Liver cancer poses a significant global public health challenge, with approximately 906,000 new cases and 830,000 deaths reported in 2020. Ranking as the sixth most prevalent cancer worldwide, it stands as the third leading cause of cancer-related mortality [1,2]. Hepatocellular carcinoma, constituting 90% of all liver cancer cases, emerges as the predominant type. The lack of effective treatments for advanced-stage tumors contributes significantly to the unfavorable prognosis associated with hepatocellular carcinoma [3]. Established therapeutic modalities, including chemotherapy, surgery, and immunotherapy, demonstrate efficacy in managing liver cancer; however, the pursuit of novel therapeutic alternatives remains imperative. Natural substances offer promise in enhancing therapeutic impact while reducing systemic toxicity and adverse effects [4].

Schiff bases, primarily characterized by the presence of imine or methylimine functional groups (–RC═N–), represent a class of organic compounds synthesized through the reaction of primary amines with aldehydes or ketones under specific conditions. In our long-standing research, we have focused on exploring the application of Schiff base compounds in anti-tumor therapy. Schiff bases exhibit a wide range of biological activities, including antifungal, antibacterial, antimalarial, anti-inflammatory, and antiviral properties. Particularly noteworthy is their significant inhibitory effect on various tumor cells, holding crucial significance in the development of novel therapeutics [5–7]. As the primary structural components of Schiff base complexes, Schiff base ligands encompass a variety of forms, including rigid, cyclic, and flexible ligands. In recent years, there has been increasing research interest in flexible ligands, mainly due to their ease of synthesis, modifiable structures, facile modification, and interchangeability, offering versatile coordination modes and demonstrating high performance with biological activity.

In our previous study, we discovered Valdien, a Schiff base ligand derived from o-vanillin, exhibiting anti-tumor effects both *in vitro* and *in vivo* [8,9]. Additionally, related studies have indicated that Valdien can suppress the proliferation of breast cancer cells (MCF-7 and MDA-MB-231) *in vitro* by modulating the Wnt1/-catenin signaling pathway [10]. Valdien inhibits proliferation and induces apoptosis of colorectal cancer cells via upregulating tumor suppressor activity of p53 to inhibit Wnt/β-catenin signal pathway [11]. Furthermore, our research has identified the anti-tumor properties of H2Valdien, along with its protective effects against chemotherapy-induced liver damage [12]. Despite the advantage of small molecular weight of H2Valdien ligand, its insolubility in water, necessitating dissolution in dimethyl sulfoxide, limits its pharmaceutical applications. To overcome this challenge, our research group has undertaken the modification of the H2Valdien ligand, successfully synthesizing water-soluble derivatives of H2Valdien.

Through our investigations, we have found that H2Valdien derivatives can significantly inhibit the proliferation and migration of liver cancer cells. Additionally, we provide evidence for the first time suggesting that H2Valdien derivatives can induce epithelial–mesenchymal transition (EMT) in liver cancer cells by modulating Hedgehog (Hh) signaling pathways.

## 2 Materials and methods

### 2.1 Cell culture and reagents

The human hepatocellular carcinoma cell line HepG2 was kindly donated by the laboratory of Gansu University of Traditional Chinese Medicine, and the cell lines Huh-7 and SMMC-7721 were kindly donated by the laboratory of the Second Hospital of Lanzhou University. HepG2, Huh-7, and SMMC-7721 human hepatocellular carcinoma cells were cultured in Dulbecco's modified eagle medium (DMEM) (Shanghai Basalmedia Technologies Co., Ltd) with 10%  fetal bovine serum (FBS), 100 U/mL penicillin, and 100 μg/mL streptomycin at 37°C in a 5% CO_2_ humidified atmosphere. The H2Valdien derivative, provided by Professor Song Pengfei (Northwest Normal University, China), was used in the study. Heat-inactivated FBS was supplied by Sijiqing Company Ltd, China. The Annexin V-PE apoptosis detection kit was purchased from Beyotime Institute of Biotechnology (Nanjing, China). Antibodies targeting EMT-related proteins (E-cadherin, N-cadherin, Vimentin) and Hh signaling pathway proteins (Smo, Shh) were obtained from Proteintech Group Inc., Chicago, IL, USA. The Hh signaling pathway inhibitor, Cyclopamine, was sourced from Abmole Bioscience Inc., Houston, USA.


**Declarations:** Hepatoma carcinoma cells HepG2, Huh-7, and SMMC-7721 derived from Gansu Provincial People’s Hospital Central Laboratory Cell Bank.

### 2.2 Cell counting kit-8 (CCK-8) assay

Logarithmic phase HepG2, Huh-7, and SMMC-7721 cell suspensions (1 × 10^4^ cells/mL) were seeded in 96-well plates. After 24 h, medium replacement occurred, and H2Valdien derivatives (final concentrations: 0, 5, 10, 20, and 40 mg/L) were introduced. Following 24, 48, and 72 h of continuous culture, 10 μL CCK-8 (Abmole Bioscience Inc., Houston, USA) solution was added to each well. Post a 2 h incubation, absorbance at 450 nm was measured using a microplate reader. Cell proliferation rates (%) were calculated as (A drug group/A blank control group) × 100%, providing a precise quantification of H2Valdien derivatives’ impact on HepG2, Huh-7, and SMMC-7721 cell proliferation with scholarly acumen.

### 2.3 Wound healing assay

HepG2, Huh-7, and SMMC-7721 cells were trypsinized, resuspended in serum-free medium, and plated on six-well plates. Following an overnight incubation to achieve confluency, linear wounds were meticulously created in the cell monolayer using pipette tips. After removing debris, cells were washed to refine wound margins. H2Valdien derivatives (5, 10, 20, and 40 mg/L) were applied for incubation. Wounds were observed and photographed after 48 h of incubation using a light microscope (Olympus Life Science, Tokyo, Japan) at 40× magnification. The proportion of healed area was quantified using the formula: percent mobility rate = migrated cell surface area/total surface area × 100. This methodological approach provides a rigorous assessment of cell motility in response to H2Valdien derivatives, contributing to a comprehensive understanding of their impact on HepG2, Huh-7, and SMMC-7721 cellular dynamics.

### 2.4 Transwell assay

The undersurface of the Transwell apparatus (Corning Costar, Corning, NY, USA) was precoated with Matrigel (Corning Costar, Corning, NY, USA), diluted fivefold with basal media. Cells were seeded in the upper chamber at a density of 8 × 10^3^ cells per well in 200 μL of serum-free DMEM medium containing H2Valdien derivatives (5, 10, 20, and 40 mg/L). The lower compartment was filled with 600 μL of DMEM medium supplemented with 10% FBS, serving as a chemoattractant. After a 48 h incubation, cells in the lower chamber were fixed with methanol and stained with 0.5% (w/v) crystal violet. Following removal of cells from the top layer, imaging of cells on the migrating layer was conducted using an inverted microscope (Olympus Life Science, Tokyo, Japan) at a magnification of ×200. Utilizing IMAGE-J software (Version 1.53r21, Bethesda, MD, USA), data were collected from nine randomly selected fields for quantification of attached and invaded cells. The average cell count per field of vision was then calculated, providing a rigorous and quantitative evaluation of the impact of H2Valdien derivatives on cell invasion dynamics.

### 2.5 Annexin V-FITC/PI double staining assay

HepG2 and Huh-7 cell suspensions (1 × 10^4^ cells/mL) were plated at 500 μL per well in a six-well cell culture plate. After 24 h, the culture medium was replaced, and H2Valdien derivatives at concentrations of 0, 5, 10, 20, and 40 mg/L were introduced for intervention. Subsequently, cells were trypsinized (without ethylene diamine tetraacetic acid addition) and centrifuged (2,000 revolutions per minute for 5 min) after 48 h of continuous culture. Flow cytometric analysis of apoptosis levels was conducted following cell washing with phosphate buffered saline (PBS) solution and processing according to the Annexin V-FITC/PI kit operating instructions. This rigorous methodology ensures precise assessment and quantification of apoptotic events induced by varying concentrations of H2Valdien derivatives in HepG2 and Huh-7 cells.

### 2.6 TUNEL assay

TUNEL staining was used to detect apoptotic cells in liver cancer cells, and according to the manufacturer’s recommendations. A fluorescent microscope was used to observe the apoptotic cells. TUNEL-positive cells emit fluorescence signals under a fluorescence microscope, observed using suitable filter sets. Images are randomly captured to ensure representative data. TUNEL-positive cells are quantified within the images, and the total cell count in the same fields is determined, often with nuclear staining. The apoptosis percentage is calculated by dividing the TUNEL-positive cell count by the total cell count and multiplying by 100. Data from multiple fields are averaged, and statistical analysis is performed to assess experimental reproducibility and significance.

### 2.7 Western blotting analysis

HepG2 and Huh7 cells (1 × 10^5^ cells/well) were treated with H2Valdien derivatives (5, 10, 20, and 40 mg/L) for 48 h at various concentrations. For western blot analysis, cells were isolated, cold PBS-rinsed, and lysed at 4°C in radio immunoprecipitation assay buffer (Beyotime, China) containing 1% phenylmethanesulfonyl fluoride. Protein concentration was determined using bicinchoninic acid testing (Solarbio, Beijing, China). Subsequently, the samples were mixed with loading buffer and boiled at 100°C for 10 min. Proteins were separated on a 10% sodium dodecyl sulfate-polyacrylamide gel electrophoresis and transferred to polyvinylidene fluoride membranes. After blocking with 5% skim milk for 2 h, membranes were incubated overnight at 4°C with primary antibodies. Following this, membranes underwent a second incubation with secondary antibodies at room temperature for an additional hour before enhanced chemiluminescence reaction was employed for blot detection. This meticulous western blotting procedure ensures accurate analysis of protein expression levels in response to H2Valdien derivatives, contributing to a comprehensive understanding of their molecular impact on HepG2 and Huh7 cells.

### 2.8 Quantitative reverse transcription PCR (qRT-PCR)

Total RNA was extracted from HepG2 cells treated with H2Valdien derivatives (5, 10, 20, and 40 mg/L) for 48 h using TRIzol (Thermo Fisher Scientific, Waltham, MA, USA). For cDNA synthesis, 2 µg of total RNA underwent reverse transcription with a cDNA Synthesis SuperMix for qRT-PCR (Yeasen, Shanghai, China). qRT-PCR was conducted to assess the expressions of E-cadherin, N-cadherin, and vimentin using SYBR Green PCR Master Mix (Yeasen, Shanghai, China). The qRT-PCR program settings included an initial denaturation for 5 min at 95°C, followed by denaturation for 10 s at 95°C and annealing for 30 s at 60°C for 40 reaction cycles. GAPDH served as a control gene, and data analysis was performed using the 2^−ΔΔCq^ method. Table 1 provides the primer sequences employed in this study. This methodological approach ensures robust quantification and analysis of gene expression profiles in response to H2Valdien derivatives in HepG2 cells.

**Table 1:** Primer sequences created in RT-qPCR

Primer name	Primer sequence (5ʹ–3ʹ)	Products (bp)
GAPDH		168
Forward	GGAAGCTTGTCATCAATGGAAATC	
Reverse	TGATGACCCTTTTGGCTCCC	
E-cadherin		162
Forward	GAGAACGCATTGCCACATACAC	
Reverse	GAGCACCTTCCATGACAGACCC	
N-cadherin		294
Forward	AAGAGGCAGAGACTTGCGAAAC	
Reverse	TGGAGTCACACTGGCAAACCTT	
Vimentin		158
Forward	GGAGGAGATGCTTCAGAGAGAG	
Reverse	GGATTTCCTCTTCGTGGAGTTTC	

### 2.9 Statistical analysis

The results from each study underwent scrutiny via one-way analysis of variance (ANOVA) and are presented as mean ± standard deviation. Statistical analyses were conducted using SPSS version 23 (SPSS, Inc., Chicago, IL, USA). Significance levels were determined, and differences between groups were considered statistically significant if *P* < 0.05.

## 3 Results

### 3.1 H2Valdien derivative inhibits hepatoma carcinoma cell proliferation *in vitro*

**Figure 1:**
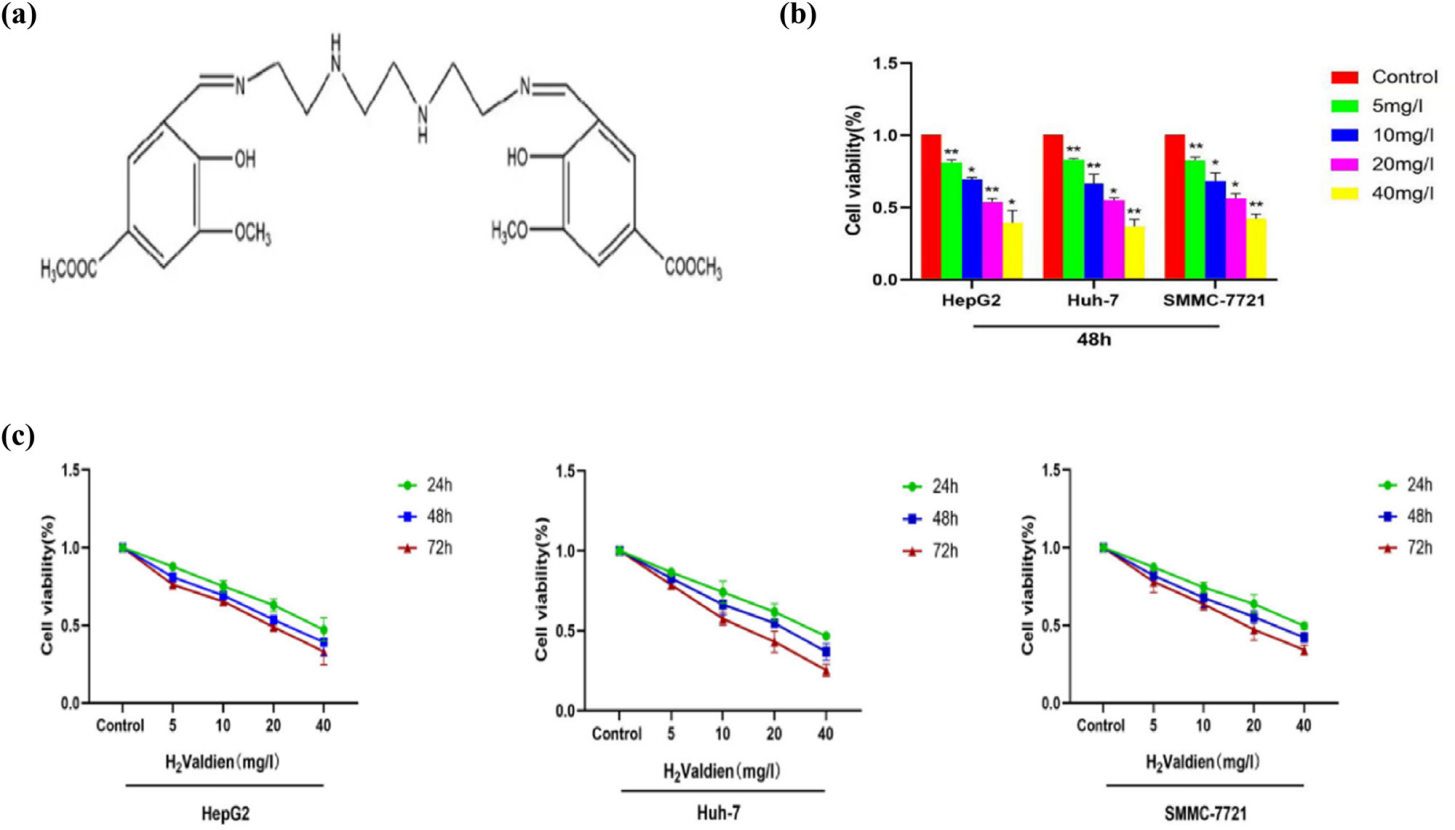
H_2_Valdien derivatives’ effects on cytotoxicity and proliferation inhibition in liver cancer cell lines. (a) Chemical structures of H_2_Valdien derivatives. (b) H_2_Valdien derivatives were utilized to treat HepG2, Huh-7, and SMMC-7721 cells at multiple doses for 24, 48, and 72 h. The CCK-8 method was used to test cell viability. (c) Different cells were treated with H_2_Valdien derivatives for 48 h to compare the inhibition of cell proliferation by H_2_Valdien derivatives and then the cell viability was measured by the CCK-8 assay. Each set of data is presented as the mean ± SD of three independent experiments. *P < 0.05, **P < 0.01.

Various concentrations (5, 10, 20, 40 mg/L) of H2Valdien derivatives (Figure 1a) were applied for 24, 48, and 72 h to hepatoma carcinoma cell lines HepG2, Huh-7, and SMMC-7721. The CCK-8 assay was employed to assess the potency of H2Valdien derivatives in inhibiting the proliferation of different hepatoma cells. Compared to control cells, the proliferation rate of hepatoma carcinoma cells exhibited a notable decrease with increasing concentrations and prolonged exposure to H2Valdien derivatives (Figure 1b). In the 48 h comparison among the three cell lines, it was observed that the inhibitory effect on proliferation was more pronounced in HepG2 cells with the application of H2Valdien derivatives (Figure 1c). These findings underscore the inhibitory impact of H2Valdien derivatives on hepatoma carcinoma cell proliferation, with distinct sensitivity variations among the cell lines.

### 3.2 H2Valdien derivatives inhibit hepatoma carcinoma cell migration and invasion

**Figure 2:**
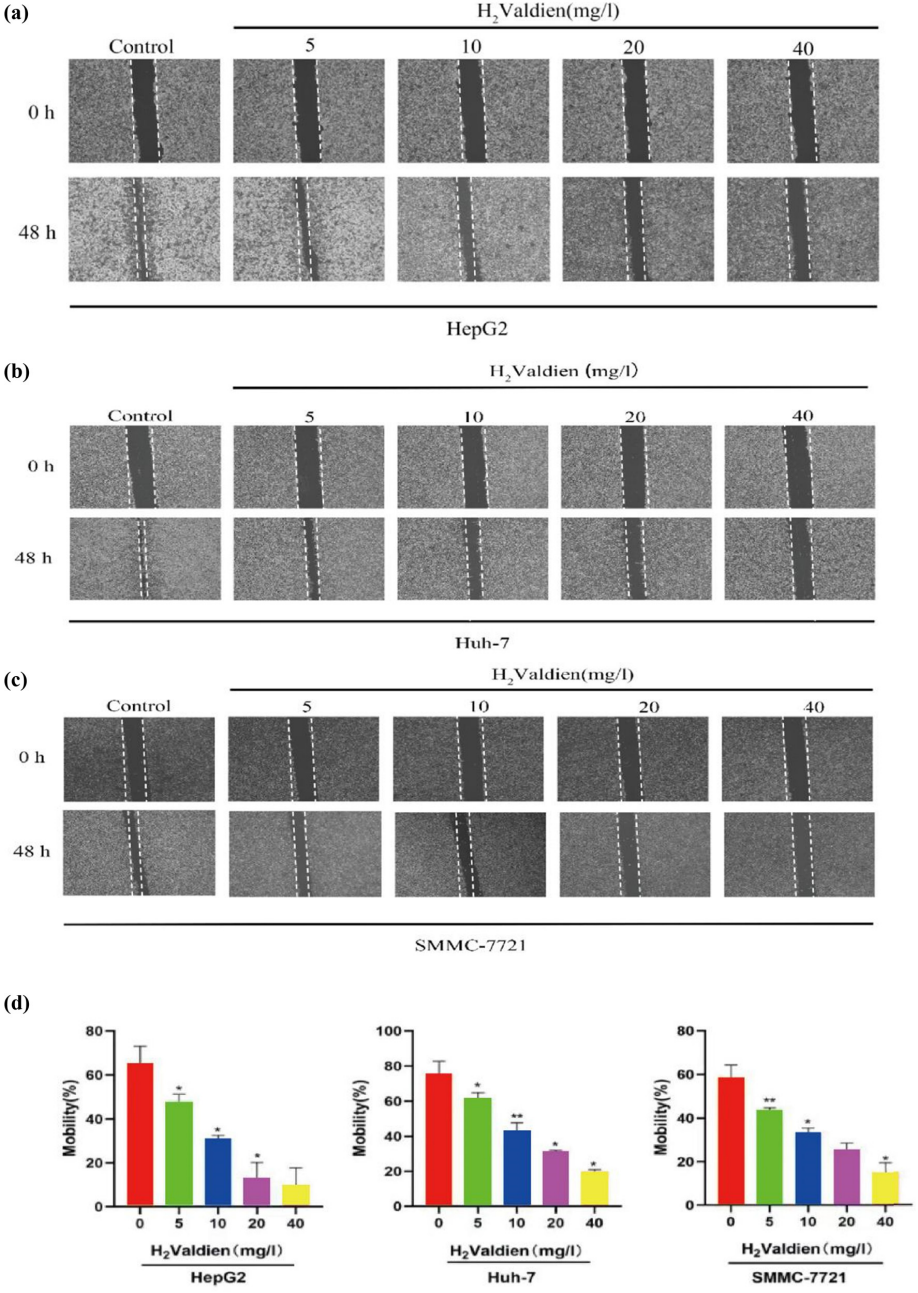
Ability of H_2_Valdien derivatives to inhibit the migrate of HepG2, Huh-7, and SMMC-7721 cells. (a)–c) H_2_Valdien derivatives were used to cultivate HepG2, Huh-7, and SMMC-7721 cells for 48 h at various doses. The mobility ratio was measured using wound healing assays after photos were taken for 0 and 48 h, respectively. (d) The scratch width was quantified to calculate the migration inhibition force of the H_2_Valdien derivatives. Each set of data is presented as the mean ± SD of three independent experiments. *P < 0.05, **P < 0.01.

**Figure 3:**
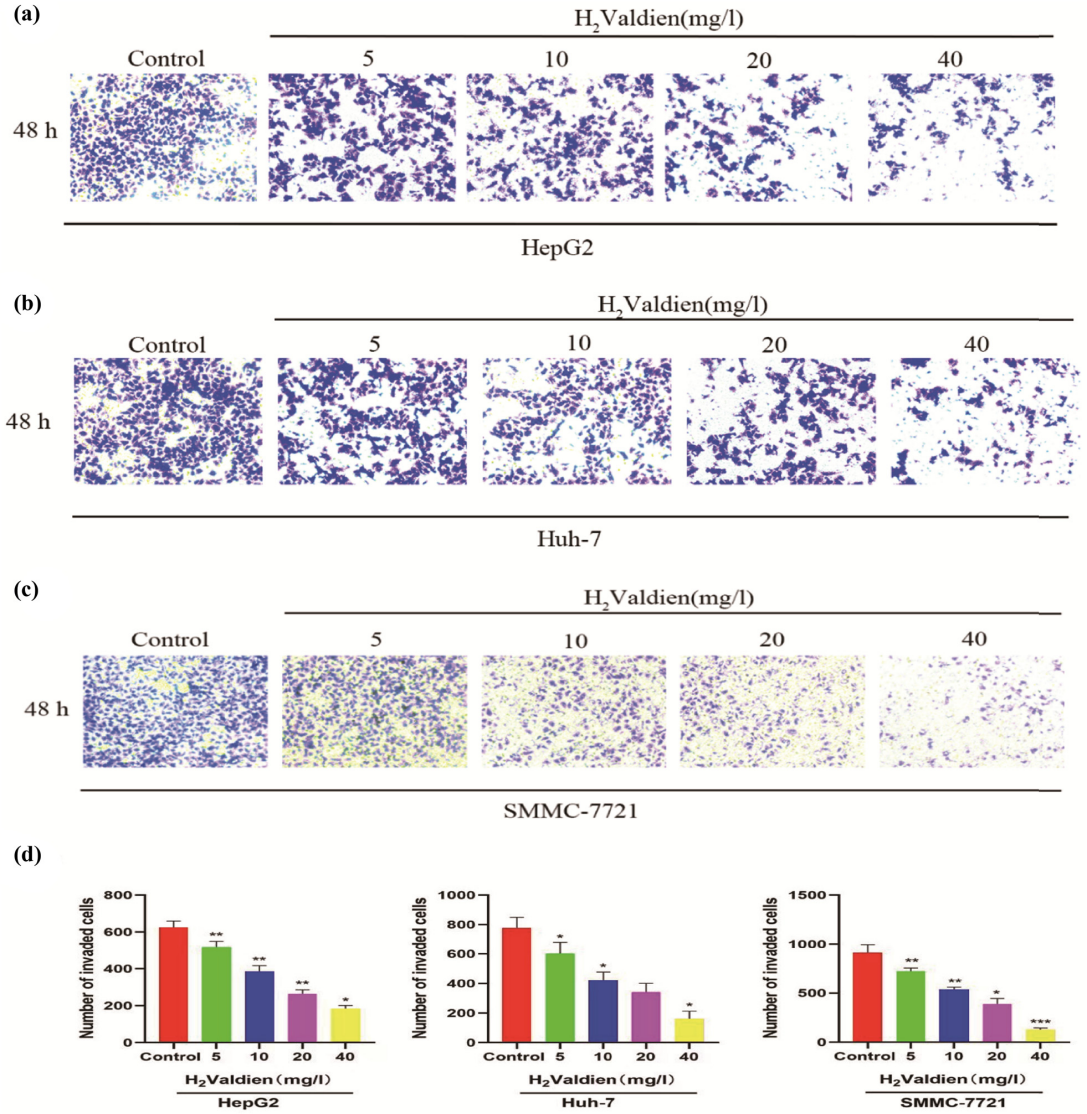
Ability of H_2_Valdien derivatives to inhibit the invasion of HepG2, Huh-7, and SMMC-7721 cells. (a)–(c) Following treatment of HepG2, Huh-7, and SMMC-7721 cells with different concentrations of H_2_Valdien derivatives for 48 h, invasion inhibition capacity was verified by the number of invaded cells. (d) The number of invaded cells was quantified. Each set of data is presented as the mean ± SD of three independent experiments. *P < 0.05, **P < 0.01.

In the context of the wound healing assay, the inhibitory effects of H2Valdien derivatives on hepatoma carcinoma cell migration were assessed by observing changes in scratch width, indicating alterations in cell mobility. The results (Figure 2a–c) reveal a pronounced, dose-dependent inhibition of hepatoma carcinoma cell migration upon exposure to H2Valdien derivatives for 48 h at concentrations of 5, 10, 20, and 40 mg/L. Notably, HepG2 cells exhibited the most significant migration inhibition, with the strongest effect observed at 40 mg/L (9.632 ± 8.134%), compared to Huh-7 (19.671 ± 1.351%) and SMMC-7721 (14.844 ± 4.733%) (Figure 2d). In tandem, invasion, a critical facet of tumor proliferation and metastasis, was investigated using Transwell assays to elucidate the impact of H2Valdien derivatives. Following a 48 h treatment with 5, 10, 20, and 40 mg/L of H2Valdien derivatives, a substantial reduction in hepatoma carcinoma cell invasion was observed, consistent with the results of wound healing tests. SMMC-7721 cells exhibited heightened sensitivity and stronger invasion inhibition, with the number of invaded cells decreasing from 910.889 ± 83.898 to 184.556 ± 15.742 at the H2Valdien derivative concentration of 40 mg/L (Figure 3a–d). In summary, H2Valdien derivatives exhibit a dose-dependent reduction in both cell migration and invasion, showcasing their potential as inhibitors of critical processes associated with hepatoma carcinoma progression.

### 3.3 H2Valdien derivatives induce apoptosis in hepatoma carcinoma cells

**Figure 4:**
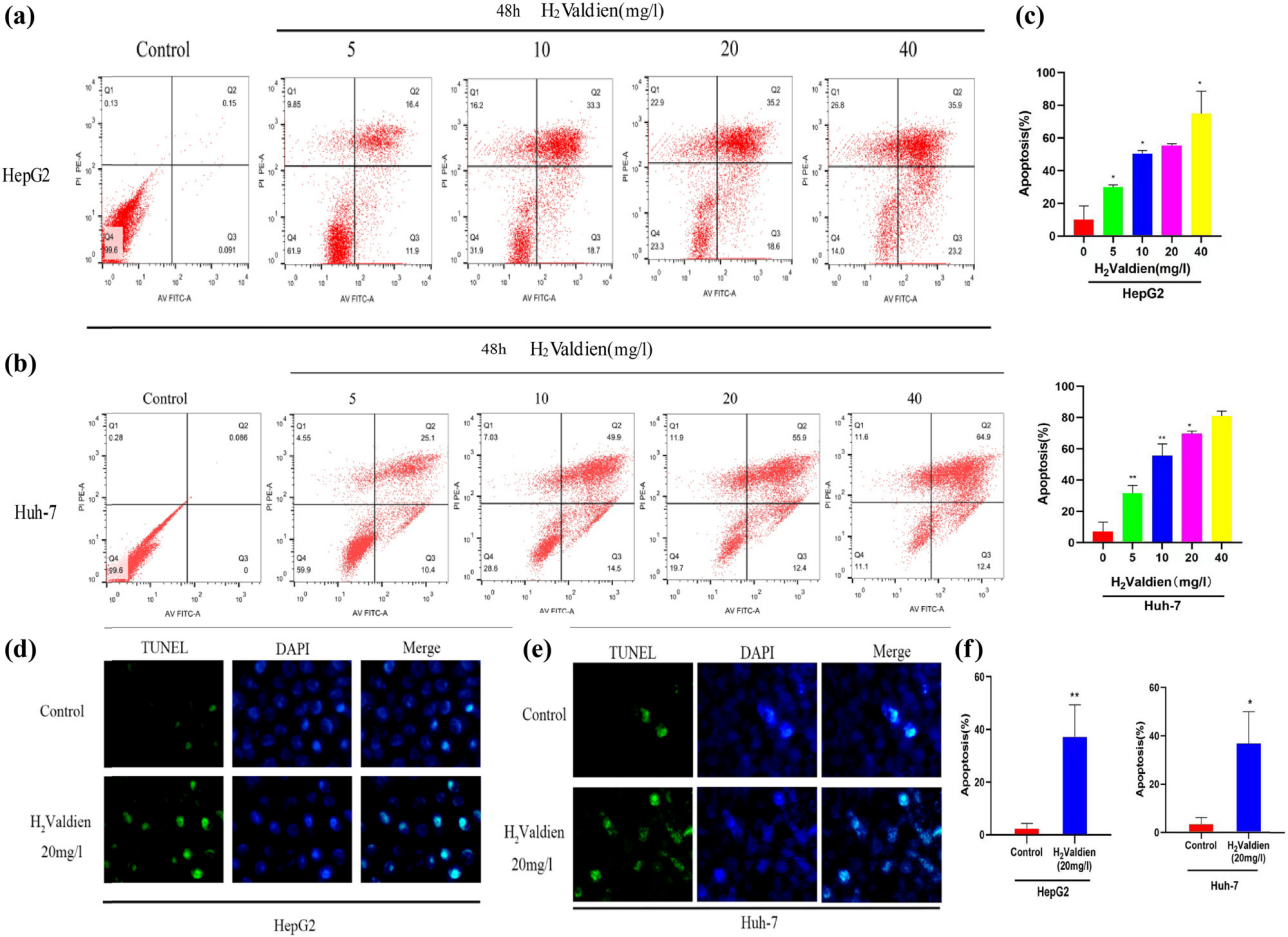
H_2_Valdien derivatives induces liver cancer HepG2 and Huh-7 cells apoptosis. (a) and (b) The HepG2 and Huh-7 cells were treated with H_2_Valdien derivatives (5, 10, 20, and 40 mg/L) for 48 h and then used Annexin V-FITC/PI double staining detected apoptosis. (c) Quantification of the sum of early apoptotic and late apoptotic cells. (d) and (e) The liver cancer cells treated with H_2_Valdien derivatives at 20 mg/L and incubation for 48 h was then measured by TUNEL staining. (f) Quantification of the number of apoptotic bodies measured in the TUNEL experiments. Each set of data is presented as the mean ± SD of three independent experiments. *P < 0.05, **P < 0.01.

The apoptotic potential of H2Valdien derivatives in HepG2 and Huh-7 cells was assessed through flow cytometry, utilizing PI and annexin V staining to distinguish between healthy, early/late apoptotic, and necrotic cells. Following a 48 h exposure to H2Valdien derivatives, both HepG2 and Huh-7 cells exhibited noteworthy apoptosis, demonstrating a dose-dependent trend (Figure 4a and b). Quantitative analysis revealed a higher induction of apoptosis in Huh-7 cells by H2Valdien derivatives (Figure 4c). To further validate the apoptotic induction, TUNEL staining was employed to detect the breakage of nuclear DNA during early apoptosis, confirming the impact of H2Valdien derivatives on hepatoma carcinoma cells. Notably, an increase in TUNEL-positive cells was observed after 48 h of treatment, indicating enhanced cellular apoptosis (Figure 4d and e). Software analysis and quantification of this assay revealed comparable apoptosis induction by H2Valdien derivatives in HepG2 and Huh-7 cells, reaching 36.850 ± 13.156 and 37.227 ± 12.088%, respectively, at a concentration of 20 mg/L (Figure 4f). These findings emphasize the significant apoptotic effect of H2Valdien derivatives in hepatoma carcinoma cells, offering promising insights into their potential therapeutic applications.

### 3.4 H2Valdien derivatives modulate EMT-associated mRNA and proteins in hepatoma carcinoma cells

In the context of cancer pathophysiology, the evolution of neoplastic cells from epithelial to mesenchymal characteristics is recognized as a critical aspect of tumor progression. This transition involves the loss of E-cadherin expression and specific cytokeratins, accompanied by the activation of markers associated with the mesenchymal state, including N-cadherin, vimentin, fibronectin, and integrins 1 and 3. To investigate whether the suppressive effects of H2Valdien derivatives on EMT are linked to specific mRNA expression, qRT-PCR was employed to evaluate mRNA levels following 48 h of incubation with H2Valdien derivatives (5, 10, 20, and 40 mg/L).

**Figure 5:**
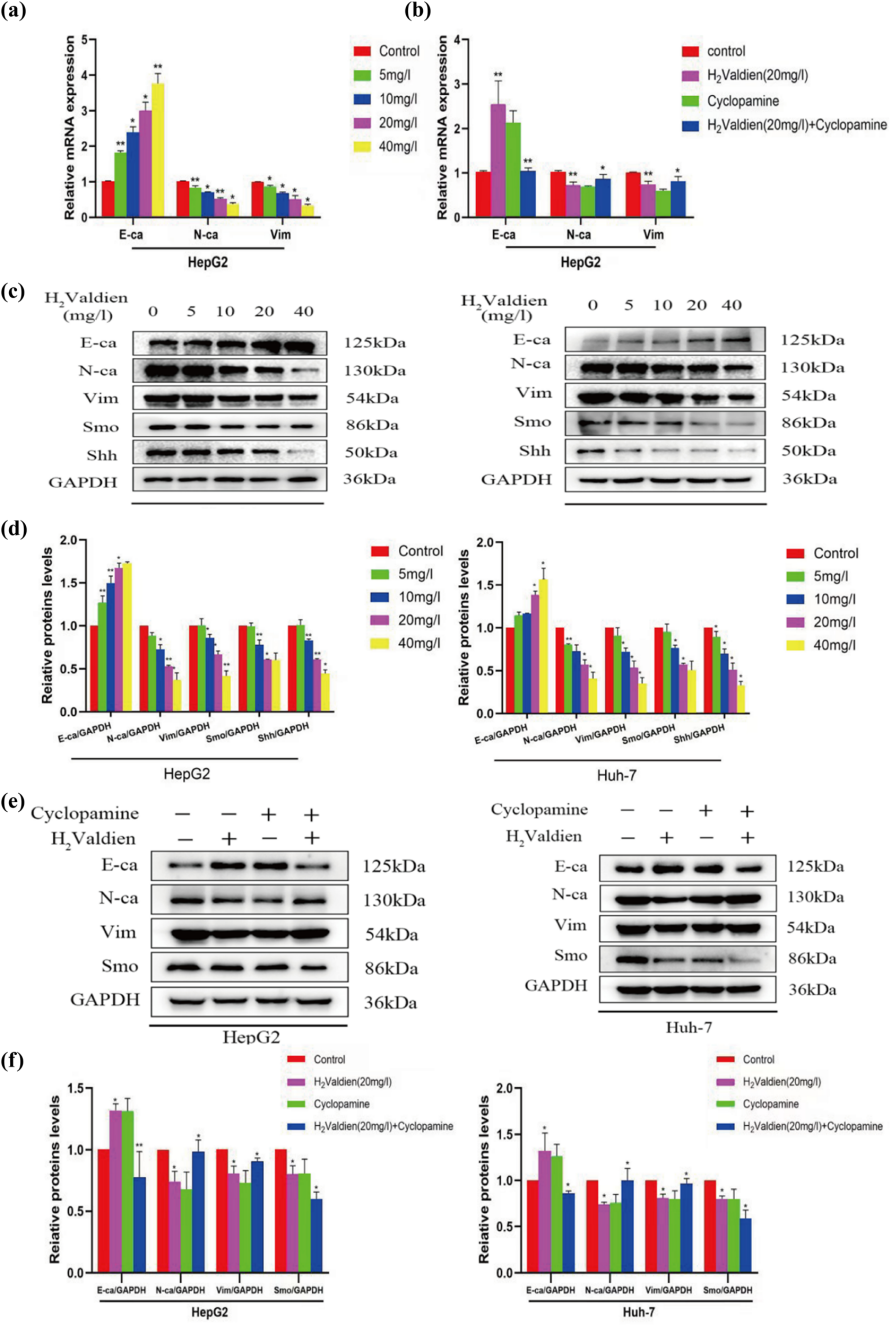
H_2_Valdien derivatives inhibited the mRNA and protein expression of Hh pathway and EMT markers in HepG2 and Huh-7 liver cancer cells. (a) EMT-associated mRNA expression was measured by RT-qPCR. (b) The corresponding mRNA expression was measured after the addition of the Hh signaling pathway inhibitors. (c) Different hepatoma carcinoma cells were treated with H_2_Valdien derivatives for 48 h, and the H_2_Valdien derivatives affected the different protein expressions in a dose-dependent manner used by western blot. (d) Quantification of protein expression after the addition of different concentrations of the H_2_Valdien derivatives. (e) Different protein expressions were detected in the same way after the addition of the signaling pathway inhibitors. (f) Quantification of the expression of the different proteins after the addition of the Hh signaling pathway inhibitors. Each set of data is presented as the mean ± SD of three independent experiments.*P < 0.05, **P < 0.01.

The results revealed a dose-dependent downregulation of N-cadherin and vimentin mRNA levels, accompanied by an upregulation of E-cadherin expression. Interestingly, the signaling pathway inhibitor Cyclopamine exhibited a similar impact on E-cadherin, N-cadherin, and vimentin expression levels (Figure 5a and b). Further exploration through western blot analysis elucidated the influence of H2Valdien derivatives on key EMT-associated proteins in HepG2 and Huh7 cells. After a 48 h incubation with varying concentrations of H2Valdien derivatives, a significant upregulation of E-cadherin expression and a noteworthy downregulation of N-cadherin and vimentin expression were observed in a dose-dependent manner (Figure 5c and d). These findings underscore the inhibitory effect of H2Valdien derivatives on EMT progression in hepatoma cells, highlighting their potential as regulators of crucial molecular processes associated with tumor metastasis.

### 3.5 H2Valdien derivatives inhibit EMT in hepatoma cells via the Hh signaling pathway

The Hh signaling pathway plays diverse roles in cancer development and progression, influencing tumor growth, proliferation, and invasion through various mechanisms. Abnormal activation of the Hh signaling pathway is implicated in these processes, making it a potential target for anti-cancer therapies. The efficacy of H2Valdien derivatives in inhibiting both the Hh signaling pathway and EMT was investigated by exposing hepatoma carcinoma cells to different concentrations of H2Valdien derivatives for 48 h. Protein analysis revealed that Smo and Shh, key components of the Hh signaling pathway, exhibited significantly lower levels in the H2Valdien derivative groups compared to the control groups (Figure 5e and f). To validate the role of H2Valdien derivatives in the Hh signaling pathway, inhibitors of this pathway were introduced, leading to a reverse verification. The inhibitor groups demonstrated an increase in E-cadherin expression, coupled with a decrease in N-cadherin and vimentin compared to groups without signaling pathway inhibitors (Figure 5e and f). Collectively, these results suggest that H2Valdien derivatives inhibit EMT in hepatoma carcinoma cells through the modulation of the Hh signaling pathway, emphasizing their potential as therapeutic agents targeting key molecular pathways involved in cancer progression.

## 4 Discussion

The extensive expansion of Schiff bases, including various organometallic compounds and aspects of bioinorganic chemistry, is notable in recent research [13]. Under specific conditions, primary amines can react with aldehydes or ketones to form Schiff bases [7,14]. In the long-term research of our group, we have focused on exploring the application of Schiff bases in antitumor therapy. Previous studies have demonstrated that Valdien, which belongs to the Schiff base family, has significant antitumor effects against human non-Hodgkin’s lymphoma. However, the poor water solubility of Valdien limits its clinical application. Therefore, we synthesized Schiff base ligands, designed a novel ligand (H2Valdien) with high water solubility, and investigated its antitumor effect and potential mechanism of action against hepatocellular carcinoma.

To assess the cytotoxicity of H2Valdien derivatives on HepG2, Huh-7, and SMMC-7721 cells, a CCK-8 assay was conducted. Results indicate that H2Valdien derivatives inhibit hepatoma cell proliferation in a dose- and time-dependent manner, with stronger cytotoxic effects observed on HepG2 cells.

A significant portion of cancer-related deaths is attributed to tumor metastasis, where cancer cells spread from a primary site to distant organs [15]. The treatment of cancer often involves the use of both antimetastatic drugs and cytotoxic chemotherapy [16]. In this experiment, the impact of H2Valdien derivatives on the metastasis and invasion of hepatoma carcinoma cells were assessed through wound healing assays and transwell assays. The results indicate that H2Valdien derivatives dose-dependently inhibit the migration and invasion of hepatoma carcinoma cells.

Cellular apoptosis, a crucial process in maintaining homeostasis, plays a significant role in tumor development and is dysregulated in cancer [17,18]. Our study employed flow cytometry to measure early and late apoptosis in HepG2 and Huh-7 cells treated with H2Valdien derivatives. The results revealed that H2Valdien derivatives induced apoptosis in liver cancer cells, with an increasing number of early and late apoptotic cells as concentrations of the derivatives increased. Additionally, a TUNEL assay confirmed the induction of apoptosis by H2Valdien derivatives, supporting the findings of annexin V-FITC/PI assays.

EMT, a conserved pathway in development, enhances cancer cell motility, invasion, and resistance to apoptosis, contributing to carcinogenesis [19]. In EMT, the expression of the epithelial marker E-cadherin decreases or is absent, while N-cadherin expression increases [20]. E-cadherin acts as a tumor suppressor, influencing polarity, differentiation, migration, and stem cell traits [21]. N-cadherin-positive cancers are associated with increased aggressiveness, and it can override the suppressive function of E-cadherin [22]. Vimentin, a component of the intermediate filament family, is widely expressed in normal mesenchymal cells, maintaining cellular integrity and providing stress resistance [23].

The study revealed that H2Valdien derivatives exerted a dose-dependent inhibition of EMT in HepG2 and Huh-7 cells. This inhibitory effect was evidenced by a decrease in mesenchymal markers N-cadherin and Vimentin, along with an increase in the epithelial marker E-cadherin.

The Hh signaling pathway, comprising ligands such as Sonic Hh (Shh), Indian Hh (Ihh), and Desert Hh (Dhh), is initiated by the interaction of Hh receptors [24]. Certain cancer-causing forms of Shh are implicated in various cancers, including basal cell carcinomas, prostate adenocarcinomas, esophageal and stomach cancers, and pancreatic cancer [25]. In addition to activating the Gli transcription factors, Smo plays a crucial role in regulating the Hh pathway, influencing epithelial cell proliferation [26]. While primary liver cancers like cholangiocarcinoma and hepatocellular carcinoma may be influenced by Hh signals, research in liver cells is still in the early stages. Activation of the Hh receptor results in decreased quiescence/epithelial indicators and a gradual increase in myofibroblast-associated genes such as α-sma, vimentin, fibronectin, and snail [27]. The Hh signaling pathway is intricately connected to the EMT. Studies have demonstrated that activating the EMT pathway promotes metastasis through the Hh signaling pathway [28].

In summary, this study presents compelling evidence supporting the inhibitory effects of H2Valdien derivatives on the proliferation of liver cancer cells *in vitro*. The findings demonstrate that H2Valdien derivatives effectively hinder cell invasion, migration, and promote apoptosis while inhibiting EMT in hepatoma carcinoma cells by targeting the Hh signaling pathway. These results demonstrate the antitumor effects of H2Valdien and its possible mechanism of action, offering experimental support for their potential as treatments for liver cancer.
